# An Exploration of the Relationships Among Facial Dimensions, Age, Sex, Dominance Status, and Personality in Rhesus Macaques (*Macaca mulatta*)

**DOI:** 10.1007/s10764-019-00104-y

**Published:** 2019-09-03

**Authors:** D. M. Altschul, L. M. Robinson, K. Coleman, J. P. Capitanio, V. A. D. Wilson

**Affiliations:** 1 Department of Psychology, University of Edinburgh, Edinburgh, UK; 2 Scottish Primate Research Group, Edinburgh EH89JZ, UK; 3 Domestication Lab, Konrad Lorenz Institute of Ethology, University of Veterinary Medicine Vienna, A-1160 Vienna, Austria; 4 Language Research Center, Georgia State University, Atlanta, GA 30034, USA; 5 Oregon National Primate Research Center, Department of Comparative Medicine, Oregon Health & Science University, Portland, OR, USA; 6 Department of Psychology, University of California, Davis, CA, USA; 7 California National Primate Research Center, Davis, CA, USA; 8 Johann-Friedrich-Blumenbach Institute for Zoology and Anthropology, University of Göttingen, Göttingen, Germany; 9 Cognitive Ethology, German Primate Center, Göttingen, Germany; 10 Leibniz Science Campus Primate Cognition, Göttingen, Germany

**Keywords:** Development, Dominance, Faces, Facial morphology, fWHR, Personality, Rhesus macaques

## Abstract

Aspects of personality in nonhuman primates have been linked to health, social relationships, and life history outcomes. In humans as well as nonhuman primates, facial morphology is associated with assertiveness, aggression, and measures of dominance status. In this study we aimed to examine the relationship among facial morphology, age, sex, dominance status, and ratings on the personality dimensions Confidence, Openness, Assertiveness, Friendliness, Activity, and Anxiety in rhesus macaques (*Macaca mulatta*). We measured facial width-to-height ratio (fWHR) and lower-height/full-height ratio (fLHFH) using photographs from 109 captive rhesus macaques, which observers also assessed for dominance status and personality, and explored the associations among facial morphology, age, sex, dominance status, and personality. fWHR and fLHFH personality associations depended on age category: Assertiveness was associated with higher fWHR and fLHFH, and Confidence was associated with lower fWHR and fLHFH, but all these associations were consistent only in individuals <8 yr. of age. We found fWHR and fLHFH to not be consistently associated with sex or dominance status; compared to younger individuals, we found few associations with fWHR and fLHFH for individuals older than 8 yr., which may be due to limited sample size. Our results indicate that in macaques <8 yr. old, facial morphology is associated with the Assertiveness and Confidence personality dimensions, which is consistent with results suggesting a relationship between fWHR and trait aggression in humans and assertiveness in brown capuchins, all of which implies that fWHR might be a cue to assertive and aggressive traits.

## Introduction

In human and nonhuman primates, the face plays an important role in social communication (Calcutt et al. 2017; Parr and Waller 2006; Waller and Micheletta 2013). In some species, such as macaques (*Macaca mulatta*, *M. fuscata*), and mandrills (*Mandrillus sphinx*), facial morphology may signal fertility (Dubuc et al. 2009; Rigaill et al. 2015; Setchell et al. 2006). The face may also provide cues to health in humans (Henderson et al. 2016; Kramer and Ward 2010; Thornhill and Gangestad 2006) and rhesus macaques (Little et al. 2012). In mandrills and drills (*Mandrillus leucophaeus*), males with stronger facial color saturation tend to be higher ranking (Marty et al. 2009; Setchell et al. 2008). In rhesus macaques, male facial coloration may indicate mate quality (Dubuc et al. 2014; Waitt et al. 2003) and is linked to competition for mating opportunities (Petersdorf et al. 2017), but is not related to dominance status (Higham et al. 2013).

**Fig. 1:**
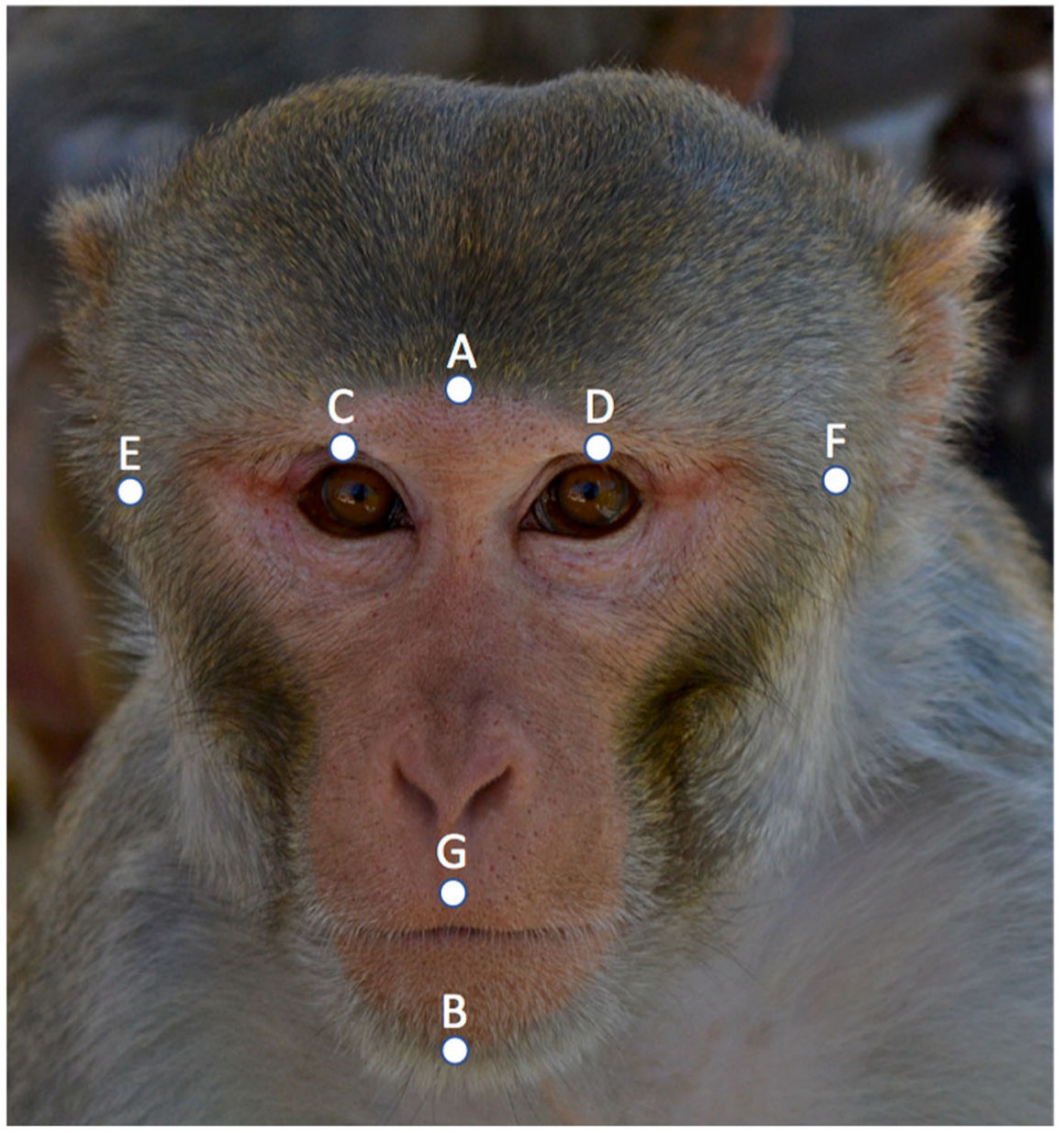
Example of measuring points used for rhesus macaque morphometric calculations. Measure for facial width-to-height ratio: (A–F)/[midpoint (C, D)–G]. Lower-height/full-heightratio: [midpoint (C, D)–B]/(A–B). We observed the rhesus macaques (*Macaca mulatta*) at the Oregon National Primate Research Center between March and June 2014, and at the California National Primate Research Center between January and April 2014.

In addition to coloration, other aspects of facial morphology may play a role in signaling social status. One such measure is the facial width-to-height ratio (fWHR), which measures bizygomatic width—the distance between the zygomatic arches—divided by superior facial height (mid-face height, i.e., nasion-prosthion) (see Fig. 1). Sexual dimorphism in fWHR was inversely related to dimorphism in canine size across 14 primate species (Weston et al. 2004), suggesting that weak dimorphism in canine size is not due to low sexual selection, but instead to selection for sexually dimorphic face width. The authors theorized that sexual dimorphism in human fWHR might be driven by female mate choice for larger cheek bones (Weston et al. 2007), a feature indicative of facial attractiveness (Cunningham 1986; Cunningham et al. 1990), although other facial features may be stronger attractiveness indicators (Mogilski and Welling 2018). Mate choice might not be the only driver of sexual dimorphism; fWHR may be a signal in male-male competition (Carré and McCormick 2008), indicating differences in dominant and aggressive tendencies that could be linked to facial morphology through underlying differences in testosterone levels (Lefevre et al. 2013; Noser et al. 2018).

Since these reports, additional studies have established that fWHR is not sexually dimorphic in humans (Kramer et al. 2012; Lefevre et al. 2012; Özener 2012); however, fWHR has been linked to aggressiveness and fighting ability in human males (Anderl et al. 2016; Haselhuhn et al. 2015; Třebický et al. 2015; Zilioli et al. 2015). Wider male faces are also perceived as being more aggressive (Alrajih and Ward 2014; Lefevre et al. 2013; Mileva et al. 2014; Stirrat and Perrett 2010), suggesting that relative face width is a social cue to aggressive and assertive behavior. While a meta-analysis of 19 studies (*N* > 4000) found support for the link between fWHR and aggression (Haselhuhn et al. 2015), a recent study in a large sample of humans (*N* > 137,000) found little evidence for any link between fWHR and self-reported behaviors such as impulsiveness (Kosinski 2017). Moreover, researchers have conducted most human studies of fWHR in western, educated, industrialized, rich, and democratic populations (Hodges-Simeon et al. 2016), which are largely socially monogamous and not representative of the diversity among human mating and societal systems.

Given these limitations and the debates within the current literature, returning to the roots of this literature and considering the role of fWHR in nonhuman primates could improve our understanding of the potential role of human facial morphology in mate choice and mate competition. Doing so provides two specific benefits. First, examining fWHR links to dominance in other primate species could allow a better appreciation of the social selection pressures that led to this association, particularly regarding sex differences. Examining links to dominance behavior among species with varying levels of fWHR dimorphism could provide a stronger ecological basis for understanding the fWHR-dominance relationship found in humans. Second, as human studies often rely on self-reported measures or proxies of dominance (Kosinski 2017), by examining nonhuman primates we can assess not only trait ratings of assertive behavior but also specifically rank, which could be more biologically relevant for understanding behavioral links to fWHR. We also examined a second facial metric, the facial lower-height-full-height ratio (fLHFH), which is introduced after fWHR.

Given the inverse relationship between canine height sexual dimorphism and fWHR dimorphism (Weston et al. 2004), brown capuchins (*Sapajus apella*) proved an ideal candidate for exploring fWHR links to dominance, given their low sexual dimorphism in canine size, similar to humans, but apparent dimorphism in fWHR (Lefevre et al. 2014; Weston et al. 2004). Findings in brown capuchins revealed that in adults of both sexes, fWHR was positively related to ratings of Assertiveness, and furthermore, fWHR was higher among alpha individuals (Lefevre et al. 2014). Whether the dimensions of the capuchin face are a social cue of assertiveness is still debated (Wilson et al. 2018). However, as has been suggested in humans, a higher fWHR could prove advantageous in male-male competition, possibly through links to stronger bite force or more robust skull structure (Lefevre et al. 2014). Research in the *Macaca* genus supports the theory that fWHR is a cue to fighting ability (Borgi and Majolo 2016). Across 11 macaque species, those with despotic dominance styles, such as in rhesus macaques, had higher fWHR in both sexes compared to more socially tolerant species, such as Tonkean macaques (*Macaca tonkeana*). These findings suggest that face width could be a signal of aggressive tendencies, particularly in females, that reduces the need for conflict within species for which escalated conflict could have serious consequences (Borgi and Majolo 2016). This result fits with findings that rhesus macaques can differentiate human faces of varying fWHR, looking longer at faces with lower fWHR (Costa et al. 2018).

In both humans (Goetz et al. 2013; Noser et al. 2018; Welker et al. 2015) and capuchins (Carré 2014) it has been suggested that the relationship between fWHR and aggressive behavior is driven by low social status, as it is significant only among low-status individuals. In brown capuchins, for example, although higher ranking individuals are typically higher in Assertiveness, correlations between fWHR and Assertiveness are significant only in non-alpha individuals (Carré 2014). Given that group members are typically aware of which is the highest ranking member of their group, there may be no need for high-ranking individuals to physically advertise dominance, as social knowledge obviates the need for this. This hypothesis proposes that low-status individuals are not necessarily low in the Assertiveness personality dimension. Assertiveness is a construct of multiple assessments of behavior, and tends to capture, among other descriptors, how independent, submissive, bullying, and manipulative (Weiss et al. 2011) an individual is.

While the current literature on facial morphology provides insights into the social role of physical features, to date, investigations of social behavior and physical features have focused on only a few species. In the current study, we expand this line of research to focus on an Old World monkey species. Given the links between fWHR and female social tolerance across the *Macaca* genus (Borgi and Majolo 2016), we aimed to explore whether this ratio is linked specifically to dominance behavior in a despotic macaque species (Thierry et al. 2004), rhesus macaques. In contrast to brown capuchins, which have low canine dimorphism and higher fWHR dimorphism (Lefevre et al. 2014; Weston et al. 2004), rhesus macaques exhibit both medium fWHR and canine height dimorphism, as well as being relatively more despotic with high levels of intragroup aggression (Thierry 2000), at least among females, factors that make them a useful comparison species for exploring links between fWHR and dominant/aggressive behaviors. This study should therefore help to build a bigger picture, across the primate lineage, of what factors might drive fWHR as a cue for dominance.

In two samples of captive rhesus macaques we studied the relationships between fWHR and two different measures of dominance: 1) hierarchical dominance status measured using normalized David’s scores and 2) a rater-derived personality dimension, Assertiveness, which assesses overall tendencies toward assertive and aggressive behavior, rather than dominance status. If rhesus macaques parallel brown capuchins, fWHR might be positively related to ratings of Assertiveness, similar to links to despotism (Borgi and Majolo 2016), with higher fWHRs found among individuals with higher social rank. Moreover, if there is a relationship between fWHR and Assertiveness, and the association is driven by low social status, then we could find a significant association between fWHR and dominance status only among individuals with lower social status, but not higher social status, as measured by normalized David’s scores (Lefevre et al. 2014). Following earlier work on personality and facial morphology (Wilson et al. 2014), we also simultaneously examined fWHR associations with ratings on five other personality dimensions, labeled Confidence, Openness, Friendliness, Activity, and Anxiety (Weiss et al. 2011), a conservative approach that allowed us to control for covariance between dimensions.

Brown capuchin males have higher fWHR than females, although this is particularly driven by mature, alpha males that have even higher fWHRs (Lefevre et al. 2014). Similarly, the link between fWHR and behavior is found predominantly among human males (Penton-Voak et al. 2001; Weston et al. 2004), so it would be consistent for fWHR associations in rhesus macaques to be driven by males. If fWHR–personality associations are driven by intrasexual selection, then given low male–male competition in rhesus, we might not find similar male-driven effects. Moreover, rhesus macaques of both sexes demonstrate significant facial skeletal growth past the point of reproductive maturity (Wang et al. 2007), so if macaques parallel capuchins, we might also see differences in the associations between skeletally mature and immature macaques (over and under age 8 yr., respectively).

Although not as widely studied as fWHR, fLHFH has previously been used as part of a masculinity index in human males to study facial attractiveness (Penton-Voak et al. 2001). Unlike fWHR, in humans fLHFH is sexually dimorphic, with males having higher fLHFH ratios than females (Penton-Voak et al. 2001), which likely captures a longer, lower face in males (Samal et al. 2007). Human jaw size is sexually dimorphic, with more prominent jaw bones being rated as more attractive in male faces (Mogilski and Welling 2018), suggesting that preference for males with larger jaws may have driven sex differences in face height. Contrastingly, fLHFH was not found to be sexually dimorphic in brown capuchin monkeys (Wilson et al. 2014), which calls into question what selection pressures could drive species differences in mate preference for jaw size. Whether, and why, this metric is sexually dimorphic in other primate species requires exploration.

Also unlike fWHR, fLHFH has been associated with higher ratings of Neuroticism and lower ratings of Attentiveness in brown capuchins of both sexes, traits strongly related to vigilance behavior (Morton et al. 2013; Wilson et al. 2014). These traits may be operationalized as one form of social status, prosocial competence (Lilienfeld et al. 2012; Wilson et al. 2014), which manifests as policing behavior (Flack et al. 2006), suggesting that facial height may also play a role in social cues. The mechanisms that underlie links between fLHFH and vigilance or policing behavior, however, are poorly understood, especially given that in capuchins, this potential cue to status does not differ by sex. It is possible that this morphological feature shares underlying variance with hormones that also drive social attentiveness. One such candidate could be testosterone, which has been implicated in vigilance to social threat (Eisenegger et al. 2011). The role of fLHFH in social behavior thus warrants further investigation, to examine whether similar effects occur in other species. As with fWHR, if rhesus macaques parallel brown capuchins (Wilson et al. 2014), we might find that ratings of lower Confidence or higher Anxiety would be most likely to be associated with higher fLHFH ratios, but we might not find a relationship between face height and David’s scores.

We additionally aimed to examine the relationship between 1) fLHFH and ratings on the personality dimensions Confidence, Openness, Assertiveness, Friendliness, Activity, and Anxiety. We also examined the relationship between 2) fLHFH and normalized David’s scores. Because we had access to a large number of independent variables, particularly personality dimensions, and wished to carry out multiple analyses within subgroups, our analyses for both fWHR and fLHFH were primarily exploratory.

## Methods

### Samples

We studied two samples of rhesus macaques: 65 (34 male) housed in 5 social groups at the ONPRC and 44 (13 male) rhesus macaques housed in 3 social groups at the CNPRC. The mean age of the ONPRC sample was 5.1 yr. (SD = 2.7, range: 2–11.6 yr) and the mean age of the CNPRC sample was 8.1 yr. (SD = 4.9, range: 1–21.1 yr).

### Personality Ratings

We used two versions of the Hominoid Personality Questionnaire (HPQ) (Weiss et al. 2009) to assess personality (see http://extras.springer.com/2011/978-1-4614-0175-9/weiss_monkey_personality.pdf): a 12-item version and the full 54-item version. Questionnaire-based approaches to personality assessment have previously demonstrated interrater and test–retest reliability (Freeman and Gosling 2010) as well as behavioral validity (Eckardt et al. 2015; Morton et al. 2013). The HPQ has been used to assess personality across multiple species of apes, Old World, and New World monkeys (Weiss 2017). Each HPQ item consists of an adjective followed by one to three sentences describing that adjective in the context of monkey behavior. For example, “FEARFUL: Subject reacts excessively to real or imagined threats by displaying behaviors such as screaming, grimacing, running away, or other signs of anxiety or distress.” Individuals familiar with the studied animals rate each monkey on each item using a 7-point Likert scale, where 1 indicates “Displays either total absence or negligible amounts of the trait” and 7 indicates “Displays extremely large amounts of the trait.” We instructed raters not to discuss their ratings with each other.

Five staff members and one researcher (LMR) rated the ONRPC macaques on the 12-item version of the HPQ. The 12-item HPQ (hereafter, short form) covered five of the six rhesus macaque personality dimensions: Confidence, Anxiety, Openness, Assertiveness (referred to as Dominance in Weiss et al. [2011]), and Friendliness. One CNPRC staff member and (LMR) rated the CNRPC macaques on the 54-item HPQ, which covered the same dimensions as the 12-item HPQ with the addition of the Activity dimension (Weiss et al. 2011). We calculated unit-weighted component scores (Gorsuch 1983) using the published six component structure (Weiss et al. 2011). Because the short form was a subset of the full HPQ, we calculated both full- and short-form component scores in the CNPRC sample, and we built full HPQ and short-form models separately where appropriate.

### Dominance Status

LMR observed 41 of the studied ONPRC macaques and all 45 CNPRC macaques using focal observations (Altmann 1974) as part of research on rhesus macaque health and welfare (see Robinson et al. 2019 for full details). Of the eight groups, six groups were observed using focal animal observation (*N* = 85; see Electronic Supplementary Material [ESM] Table SI for individual group characteristics) resulting in some missing data (e.g., normalized David’s scores) for the 24 macaques housed in the remaining 2 groups. A complete description of the observed ONPRC and CNPRC samples is available in Robinson et al. (2019).

We observed the macaques for multiple behaviors including supplant behaviors, which we defined as: focal macaque is touched by a conspecific and the focal macaque moves and conspecific may or may not take the focal macaque’s spot, or focal macaques moves in response to a conspecific’s touch. For the full ethogram see Supplementary Table #1 in Robinson et al. (2019). Across the two facilities, we observed each macaque for a mean of 224.09 ± SD 57.22 minutes. We then used these data to create directional supplant matrices for each rhesus macaque group, which we then used to calculate each macaque’s normalized David’s score (de Vries et al. 2006). We pooled both sexes into the same dominance hierarchies, and we investigated sex differences via interactions in regression models to preserve sample size, rather than splitting males and females.

### Age Categories

Significant physical changes in body size (Bernstein and Ehardt 1985) and facial morphology (Cheverud and Richtsmeier 1986) occur as rhesus macaques sexually mature and become adult. Although sexual maturity occurs at ages 3 yr. and 4 yr. in females and males respectively, skeletal maturity does not occur until age 8 in both sexes (Cheverud and Richtsmeier 1986; Wang et al. 2007). We divided our sample into younger (81 individuals <8 yr) and older (28 individuals >8 yr) subsamples to determine if the associations we investigated were unique to one age group but not the other (Wang et al. 2007). The younger age group included all individuals who may still experience developmental changes in the skeletal structure of their faces, whereas the older group contained only mature individuals who are no longer growing. In some analyses, we examined these subgroups in addition to the entire sample that encompasses both age groups.

### Facial Measurements

We photographed macaques’ faces after we completed the focal observations for each project, either while the macaques were in their social groups or during regularly scheduled sedation as part of health checks using the same photography method each time. We took multiple photographs of neutral expressions for most individuals (mean = 2.38 ± SD 1.39), up to a maximum of 7; 20 individuals were represented by only one photograph. We did not use photos if key facial features were not visible, or if the individual’s face was not parallel and ventrally head-on to the camera; we measured 238 usable photographs. We defined facial metrics with a total of seven points (Fig. 1) and we computed fWHR as the ratio of bizygomatic-width (maximum horizontal distance from the left to the right facial boundary) to upper face height (vertical distance from the midpoint of the upper lip to the highest point of the eyelids) (Lefevre et al. 2014; Wilson et al. 2014). We calculated fLHFH as the distance between the highest point of the eyelids and the lowest point of the chin divided by the length of the whole face, i.e., from brow to chin. We reassessed 24 images at random (from 21 different monkeys), i.e., 10% of the total number of face photographs, and compared them to the original assessments to evaluate the reliability of our measurements.

### Study Design and Statistical Analyses

With these demographic, personality, and facial morphology data we carried out a series of exploratory regression analyses. These analyses were exploratory because 1) that was an analytical choice we made before carrying out any analyses: we would investigate many variables and interactions of potential interest, and 2) it was a necessity: macaque personality structure is not perfectly analogous to capuchin or human personality structure (Weiss et al. 2011). Direct predictions cannot be made, particularly when on the human side of the field, the existence of associations between fWHR and personality is contested (Haselhuhn et al. 2015; Kosinski 2017). Our aim was thus to carry out exploratory analyses and generate rhesus macaque specific hypotheses, which can then be tested in future work. Although this study was exploratory, we nevertheless planned the analytic approach described in the text that follows in advance, although we did not formally preregister our analyses.

We assessed reliability of facial measurements using three metrics. Cronbach’s alpha (*α*) is a test of internal consistency, that is, if two assessments are measuring the same thing. In this case the assessments are the originals and retests of a given face photograph by the same rater. Though widely used, *α* overestimates reliability, so we supplemented it with the Pearson product-moment correlation coefficient (*r*) and Guttman’s lambda 6 reliability (Revelle and Zinbarg 2009). fWHR and fLHFH were calculated for both the original and retest measurements, then test–retest reliability was compared using these statistics. We also assessed the reliability of the individual point placements on macaques’ faces.

We primarily assessed the relationships between variables using linear mixed models (Bates et al. 2014), and the assumptions made by these models were checked for violations (Bolker et al. 2009). Where possible, we estimated bootstrapped 95% confidence intervals, but when this was not possible, we computed profile or Wald intervals. We conducted all analyses in the R programming language (version 3.4.2).

Our exploratory approach followed a model building procedure that minimized individual steps; we formulated steps in terms of successive sets of variables explaining variance over and above the preceding sets. Variables that did not improve model fit were not retained to avoid collider bias (Cole et al. 2009). First, we examined age, age^2^, and age^3^ associations with our outcome variables, fWHR and fLHFH. Age^2^ and age^3^ were included to assess nonlinear age changes. Second, we added sex and a sex × age interaction. Third, we added normalized David’s scores and its interaction with sex. Fourth, we added personality variables, in separate models for the full and short-form HPQ, as using only the short-form HPQ allowed us to enlarge the sample. Finally, for fWHR, at step 4 we also examined a David’s score × Anxiety interaction. At each step, we retained variables if they were significant or if overall model fit was improved. After step two analyses of age associations across the whole sample, we carried out subsequent analyses simultaneously in the full sample, younger subsample, and older subsamples using the same variables; if we retained a variable in one sample it was retained in all for ease of comparability. fWHR and fLHFH were analyzed in separate models but following the same process.

#### Data Availability

Data are available in the online supplemental files at the *International Journal of Primatology* website.

### Ethical Note

This project was noninvasive and purely observational and complied with the US Animal Welfare Act. The Institutional Animal Care and Use Committees at the Oregon National Primate Research Center (ONPRC) and California National Primate Research Center (CNPRC) approved these studies. Ethical approval was given by the ONPRC, CNRPC, and University of Edinburgh’s Biological Services Unit, AWERB OS2-14 and A3433-01. The authors declare that they have no conflict of interest.

## Results

### Reliability of Facial Measurements

All statistics indicated that our ratings of faces were reliable from one individual to the next (Table I). We also found only small deviations in placement of points A through G from one assessment to the next (ESM Table SII). We describe the complete details of our facial reliability analyses in the online supplement, along with the correlation matrix for all variables of interest (ESM Table SIII).

### Facial Width/Height Ratio

#### Age, Sex, and Dominance Status

The best fit model contained age, age^2^, and age^3^ (χ^2^ = 4.24, df = 7, *P* = 0.039), although none of the variables were significant on their own in the model (Table II). Building on this model by including sex and an age × sex interaction revealed a significant effect of sex, but no interaction (ESM Table SIV).

In our models of age and sex in the younger and older subsamples, we found no age × sex interaction (Table II). We found a significant effect of sex for the younger group, but not for the older group, suggesting that sex differences in face structure within the younger age group drive the overall effect.

Retaining sex, we added normalized David’s scores to our models. Normalized David’s score was not associated with fWHR in the full sample or subsamples (Table III). Normalized David’s scores also interacted with sex in similar models, but in these models neither David’s scores nor the interaction was significant. We thus excluded normalized David’s scores from additional models of fWHR.

#### Personality

In a model including monkeys rated on the full HPQ, we found no personality dimensions associated with fWHR (Table IV). However, in the younger sample, we found Activity was negatively associated and Assertiveness positively associated with fWHR, while in the older sample, Assertiveness was negatively associated with fWHR, but Confidence was positively associated with fWHR.

**Fig. 2:**
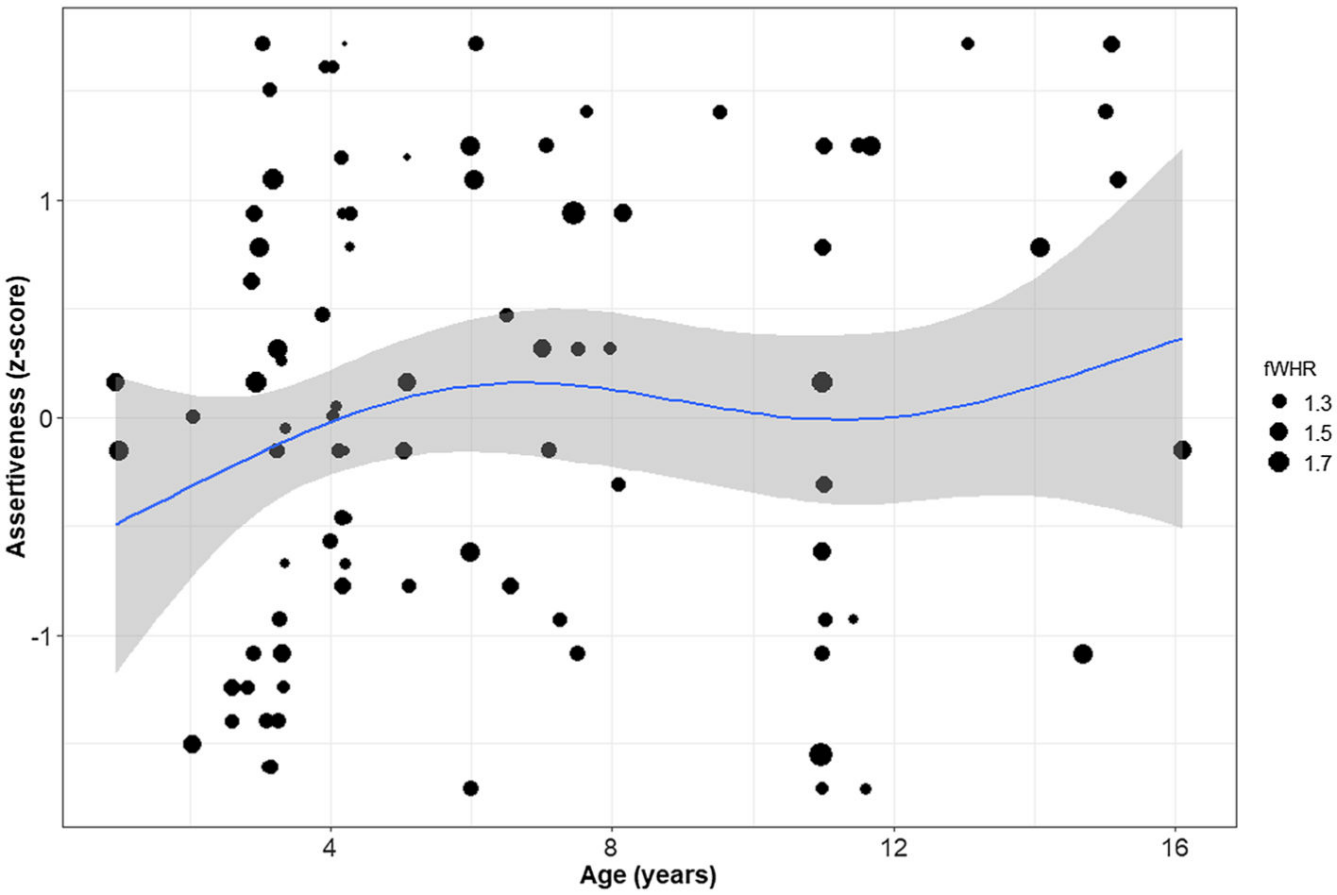
Scatterplot of age, Assertiveness, and facial width-to-height ratio (fWHR). The size of the dot represents the magnitude of an individual’s mean fWHR across ratings; the scale is in the legend. The line is a generalized additive model regression line and 95% confidence region, fit to the data for individuals of all ages. We observed rhesus macaques (*Macaca mulatta*) at the Oregon National Primate Research Center between March and June 2014, and at the California National Primate Research Center between January and April 2014.

We built similar models based on the shortened definition of four macaque personality dimensions, which allowed us to use animals from both facilities. In these models, we again found no associations between personality and fWHR that spanned the full sample. However, when we split the sample we increased discriminability within age groups, which allowed these models to detect a positive association between fWHR and Assertiveness and a negative association with Confidence, in the younger sample. We found no associations in the older sample. The positive linear association between Assertiveness and fWHR is visible in the under-8 individuals but the fit for the smaller, older group is not apparent (Fig. 2).

We were also interested in further exploring the relationship between Anxiety and fWHR, as lower ranking primates tend to experience more stress under certain social conditions and may express this through higher Anxiety (Abbott et al 2003). Thus, in post hoc analyses we built models that included all age variables, sex, dominance status, Anxiety, and the interaction of dominance status and Anxiety (Table V). The main effects of Anxiety (using the shortened questionnaire) and dominance status were significant in the full sample, as was the interaction between dominance status and Anxiety. These effects were all significant in the younger sample as well. Though none were significant in the older sample, the directions of effects were consistent in all groups. These results indicate that higher Anxiety individuals tend to have lower fWHR, and individuals that are high in dominance status and high in Anxiety or low in dominance status and low in Anxiety have higher fWHR. We examined models that used the full HPQ subsample, but we found no significant effects.

### Facial Lower-Height/Full-Height Ratio

#### Age, Sex, and Dominance Status

We followed the same analytic strategy with fLHFH as with fWHR. Modeling the effects of age, age^2^, and age^3^, on fLHFH, we again found that the best fit model contained all three age variables (χ^2^ = 12.4, df = 7, *P* < 0.001). Unlike for fWHR, the associations between the age variables and fLHFH were significant (Table VI). We also split the sample into younger and older monkeys at this stage; the age effects were not significant in either subsample, but were in the same direction in all variations.

Adding sex and an age × sex interaction to the best fit model, we found no effect for either sex (*B* = −0.015, CI: [−0.044, 0.016]) or the interaction (*B* = −0.011, CI: [−0.047, 0.024]). This held true in the younger (sex: *B* = 0.034, CI: [−0.092, 0.023]; interaction: *B* = −0.048, CI: [−0.127, 0.044]) and older (sex: *B* =0.034, CI: [−0.202, 0.274]; interaction: *B* = −0.031, CI: [−0.258, 0.192]) subsamples, so we did not retain sex in subsequent models of fLHFH. With normalized David’s scores, we followed the same process as we did with our fWHR models, and similarly, found no association between dominance status and fLHFH in the full sample (*B* = 0.002, CI: [−0.005, 0.008]) or either younger (*B* =0.002, CI: [−0.005, 0.009]) or older (*B* =0.003, CI: [−0.016, 0.012]) subsamples.

#### Personality

We first modeled personality’s influence on fLHFH using the sample with full ratings for all six personality dimensions. As with our initial models of fWHR and personality, we found no associations between the variables (Table VII). We also found no association between the personality dimensions and fLHFH in the older group, but in the younger group we found a positive association between Assertiveness and fLHFH, and a negative association between Confidence and fLHFH. Using the shortened questionnaire dimensions from animals at both facilities, we also found no effects of personality across the entire sample, or in either subsample (ESM Table SVI).

## Discussion

We found facial dimensions in rhesus macaques to be related to variables of age, sex, and personality, although the nature of these relationships varied somewhat. There were no specific, consistent effects of age, age^2^, or age^3^, but their inclusion in our models did improve fit, and thus they made a meaningful contribution. In other words, although the age variables were not always significant in our models, the interdependence between these variables and the outcomes supports an age effect across the macaque lifespan (at least through the ages present in our samples), such as the differences we found between skeletally mature and immature macaques. Splitting the sample by these age groups (Bernstein and Ehardt 1985; Cheverud and Richtsmeier 1986; Wang et al. 2007) consistently made our models more interpretable, and differences we found in the associations between facial dimensions and age group suggest that changes in facial morphology may co-occur with psychological development. Table VIII summarizes our findings.

In contrast to work across the macaque genus (Borgi and Majolo 2016), which found no sex difference in fWHR across 11 macaque species, we found that females across the sample have wider faces, particularly in the younger sample of macaques. There were, however, no sex effects on fLHFH. We also found no interactions between age and sex, which contrasts with findings in capuchin monkeys, in which sex was not associated with either fWHR or fLHFH, but an age × sex interaction was found with both (Lefevre et al. 2014; Wilson et al. 2014).

We also found that fWHR was associated with aspects of personality, but in different ways depending on which age category the individual fell within. Assertiveness was negatively associated with fWHR in older monkeys (above age 8), but positively associated in younger monkeys (under age 8); contrastingly, Confidence was negatively associated with fWHR in young monkeys. Activity was also negatively associated with lower fWHR in the young macaques. The relationship between Assertiveness (item loadings from Dominant, Bullying) and fWHR in the young group reflects findings in capuchins, where Assertiveness (item loadings Bullying, Aggressive, Dominant) was positively associated with fWHR (Lefevre et al. 2014). Associations between fLHFH and personality and other variables were weaker. fLHFH was associated with Confidence and Assertiveness in the same way as fWHR, again, only in the young subsample of macaques.

It has been hypothesized that the fLHFH ratio is a potential signal of status in capuchins, conferred not through aggressive or dominant behaviors but through a policing role (Flack et al. 2006), which could explain the links to vigilant behavior in brown capuchins (Wilson et al. 2014). In rhesus macaques, the lack of a relationship in the older sample suggests that this association is not indicative of some form of social cue. That these findings for rhesus macaques do not reflect findings in brown capuchins suggests that the relationship between status and face height may differ between species. Our evidence only suggests that, at least in macaques, fLHFH is measuring the same underlying physical characteristics as fWHR. This is consistent with the correlation of *ρ* = 0.19 (Table SIII) that we calculated between fLHFH and fWHR. However, in brown capuchins the correlation was much lower, 0.02 (Wilson et al. 2014), so to determine whether fLHFH is a distinct social signal of some kind, further investigation in other primate species are warranted.

We found no relationships between dominance status (i.e., normalized David scores) and fWHR or fLHFH. Earlier theory and results from a variety of macaque species suggest that fWHR could be a cue to social rank (Borgi and Majolo 2016). Moreover, work in capuchins found an association between alpha status and fWHR (Lefevre et al. 2014). Despite finding no relationship between dominance status and fWHR, it is notable that dominance status had considerable overlap with both the Confidence and Assertiveness personality dimensions (Table SIII), which were both related to fWHR. Thus, it seems that personality alone, rather than personality as a proxy for social standing, relates to facial morphology. This runs counter to the suggestion that fWHR is a visual signal for social status, or is driven by low-status individuals (Carré 2014; Goetz et al. 2013; Welker et al. 2015), though we were unable to directly test this hypothesis with this sample.

Another possibility is that dominance status is more important as a physical cue in New World monkeys, whereas in Old World monkeys Assertiveness rather than dominance status is a stronger predictor of fWHR variance. This fits with the findings that rhesus macaques differentiate between faces based on trustworthiness (Costa et al. 2018). Rhesus macaques are “despotic” (Thierry 2000) and among the most intolerant of macaque species, whereas capuchins are known for being socially tolerant within their groups (Fragaszy et al. 2004). Differing levels of social tolerance may explain the differences in personality associations between rhesus macaques and brown capuchins. It would be informative to explore to what extent social tolerance of different species varies with differences in fWHR. If the relationship between fWHR and dominance behavior varies with social status, then one would expect to find larger differences in this ratio between alpha and non-alpha individuals in despotic species over socially tolerant species. One difficulty of this approach is that one must establish a measure that can account for not only species differences but also intraspecific sex differences in social tolerance, something that we were unable to fully address in this study.

The presence of both an Assertiveness and Confidence dimension in rhesus macaque personality structure complicates interpretations of these associations with facial morphology. Confidence is characterized by items like “fearful,” “submissive,” “cautious” – all reverse coded. Assertiveness is characterized by “dominant,” “bullying,” and “irritable.” Thus, these constructs are already measuring similar underlying processes, so a possible explanation for our results is that as younger monkeys mature, a shift in behavior changes the associations between individual features and both Assertiveness and Confidence. One such feature could be fWHR.

We found a negative association between Anxiety and fWHR. Typified by “quitting,” “anxious,” “erratic,” and the reverse coding of “cool,” Anxiety overlaps with both human and capuchin Neuroticism (Costa et al. 2017; Morton et al. 2013). Neuroticism was associated with fLHFH in capuchins, but not with fWHR. Anxiety has a low correlation with dominance status, so as with Confidence and Assertiveness, it is unlikely that dominance status confounded this association. However, the positive interaction we found between dominance status and Anxiety indicates that more dominant individuals tend to either have higher Anxiety or higher fWHR. One possible reason for this is that without the physical social signal of fWHR to support their position, more dominant individuals experience more stress and have higher Anxiety as a result.

While a growing body of literature has developed around fWHR (Borgi and Majolo 2016; Haselhuhn et al. 2015; Kosinski 2017; Lefevre et al. 2014; Weston et al. 2007) and fLHFH (Penton-Voak et al. 2001; Wilson et al. 2014), associations between these physical variables and psychological measures are understudied in juvenile and subadult primates (Hodges-Simeon et al. 2016; Welker et al. 2016; Zebrowitz et al. 2015). Our study suggests that age may influence how facial dimensions relate to personality. None of the associations between personality and our facial dimension measures spanned both age categories. As we suggested, the same underlying processes could be involved in different associations but unearthing the physiological and developmental processes involved is beyond the scope of this study.

Despite having a larger sample of macaques to work with when we analyzed the short-form data, associations between facial ratios and the shortened personality variables were often weaker. This is consistent with the finding in the field of personality psychology that fewer items generally results in poorer validity (Clark and Watson 1995). We found associations only in the younger group, which fit the data: we used the shortened questionnaire at the ONPRC and the mean age of that sample was younger than that of the CNPRC. Owing to the limitations of the shortened questionnaire, we likely only had the power to detect the same effects in the younger subsample of monkeys. However, the associations we did find between the shortened dimensions and facial ratios were consistent with our overall results.

The relationships among sex, social status, and dominance behaviors are complex and variable (Bernstein 1970; Janson 2017), and incorporating social style is a particular issue for studies of macaques because social style is derived from female behavior. Social style appears to be related to fWHR across sexes (Borgi and Majolo 2016), but without a comprehensive understanding of the male behaviors that complement tolerant or despotic female behavior, it is difficult to conclude how pressures on male social position would relate to social signals like fWHR or fLHFH.

Our sample of skeletally immature macaques was larger than our sample of skeletally mature macaques. Our overall results are more representative of the younger population, and further work is necessary to derive any solid conclusions about the associations among personality, social status, sex, and fWHR in older rhesus macaques. Our findings show that even from a young age, facial differences emerge that can be linked to personality differences.

These results are broadly consistent with previous work in capuchin monkeys and humans. In humans, fWHR is most often associated with aggression (Haselhuhn et al. 2015), though the effect is small. Rhesus macaques are a despotic, sexually dimorphic, species, and associations between facial dimensions and dominance-related traits are likely to be stronger than the relationships one might find in humans. Thus, the multiple relationships between dominance-related traits and facial measures we found in rhesus macaques suggest that associations between personality and facial morphology may have been present deep in our phylogenetic history, beyond the common ancestor of Old World and New World monkeys. However, our results are not consistent with associations found between fLHFH and personality in capuchin monkeys, suggesting in this respect, the associations among personality, behavior, and fLHFH may have diverged since the split between Old and New World monkeys.

## Supplementary Material

Supplement

## Figures and Tables

**Table I:** Reliability statistics for two facial measures

	Cronbach’s *α*	Guttman’s λ, *G6*	Pearson correlation, *r*
Facial width-to-height ratio	0.81	0.66	0.81
Lower-height/full-height ratio	0.97	0.90	0.95

Photographs were taken of rhesus macaques (*Macaca mulatto*) observed at the Oregon National Primate Research Center between March and June 2014, and at the California National Primate Research Center between January and April 2014

**Table II:** Associations between facial width-to-height ratio, age, and sex, split by age group

Variable	Younger				Older			
	*B*	95% C.I.	*B*	95% C.I.	*B*	95% C.I.	*B*	95% C.I.
Age	−0.88	[−1.85, 0.20]	**−0.93**	[**−1.99, −0.05**]	−0.59	[−4.19, 2.59]	1.26	[−5.90, 7.88]
Age^2^	2.90	[−1.90, 7.32]	3.55	[−0.35, 8.18]	0.92	[−3.19, 5.59]	−1.74	[−10.9, 8.50]
Age^3^	−2.72	[−8.34, 3.48]	−3.88	[−9.72, 1.10]	−0.43	[−2.27, 1.19]	0.76	[−3.59, 4.78]
Sex			**−0.12**	**[−0.21, −0.04]**			0.20	[−0.48, 0.84]
Sex × Age			−0.05	[−0.18, 0.07]			−0.20	[−0.81, 0.40]

We fitted two models each to the younger and older data, the first without sex and an age × sex interaction, and a second model with. Bold indicates estimates whose confidence interval did not overlap with 0. We observed rhesus macaques (*Macaca mulatta*) at the Oregon National Primate Research Center between March and June 2014, and at the California National Primate Research Center between January and April 2014

**Table III:** Mixed models of facial width-to-height ratio, sex, dominance status, and age variables

Variable	All individuals		Younger		Older	
	*B*	95% C.I.	*B*	95% C.I.	*B*	95% C.I.
Age	−0.19	[−0.44, 0.06]	−0.84	[−1.85, 0.01]	−0.59	[−4.08, 2.89]
Age^2^	0.37	[−0.13, 0.87]	3.31	[−0.47, 7.67]	1.17	[−3.40, 5.75]
Age^3^	−0.20	[−0.47, 0.07]	−3.88	[−9.33, 0.94]	−0.63	[−2.48, 1.21]
Sex	**−0.07**	**[−0.14, −0.02]**	**−0.10**	**[−0.15, −0.05]**	0.17	[−0.17, 0.50]
Dominance status	−0.01	[−0.02; 0.01]	0.01	[−0.02, 0.01]	−0.03	[−0.08, 0.02]

Bold indicates estimates whose confidence interval did not overlap with 0. We observed rhesus macaques (*Macaca mulatta*) at the Oregon National Primate Research Center between March and June 2014, and at the California National Primate Research Center between January and April 2014

**Table IV:** Mixed models of facial width-to-height ratio, personality, age, and sex variables

Variable	All individuals		Younger		Older	
	*B*	95% C.I.	*B*	95% C.I.	*B*	95% C.I.
Age	−0.21	[−0.56, 0.13]	−0.06	[−1.61, 1.35]	−2.19	[−5.65, 1.30]
Age^2^	0.41	[−0.25, 1.05]	−0.36	[−7.33, 6.94]]	3.20	[−1.25, 7.60]
Age^3^	−0.22	[−0.56, 0.13]	0.30	[−9,87, 9.91]	−1.37	[−3.07, 0.36]
Sex	−0.01	[−0.12, 0.11]	0.11	[−0.11, 0.34]	0.13	[−0.21, 0.46]
Confidence	0.03	[−0.14, 0.19]	−0.15	[−0.35, 0.05]	**0.37**	**[0.03, 0.72]**
Openness	0.06	[−0.08, 0.19]	0.07	[−0.10, 0.27]	0.29	[−0.01, 0.59]
Assertiveness	−0.03	[−0.21, 0.14]	**0.23**	**[0.05, 0.46]**	**−0.44**	**[−0.77, −0.11]**
Friendliness	0.01	[−0.07, 0.08]	0.06	[−0.12, 0.22]	−0.09	[−0.27, 0.09]
Activity	−0.10	[−0.2, 0.01]	**−0.22**	**[−0.44, −0.01]**	−0.13	[−0.28, 0.02]
Anxiety	0.05	[−0.05, 0.16]	0.00	[−0.25, 0.22]	0.10	[0.05, 0.26]

Removing age variables from the model of younger macaques did not substantively change the results. Bold indicates estimates whose confidence interval did not overlap with 0. We observed rhesus macaques (*Macaca mulatta*) at the Oregon National Primate Research Center between March and June 2014, and at the California National Primate Research Center between January and April 2014

**Table V:** Mixed models of facial width-to-height ratio and Anxiety by dominance status interactions

Variable	All individuals		Younger		Older	
	*B*	95% C.I.	*B*	95% C.I.	*B*	95% C.I.
Age	−0.19	[−0.43, 0.06]	**−1.00**	**[−1.97, −0.17]**	−0.79	[−4.12, 2.55]
Age^2^	0.38	[−0.10, 0.86]	**4.14**	**[−0.50, 8.40]**	1.52	[−2.88, 5.92]
Age^3^	−0.22	[−0.48, 0.04]	**−5.04**	**[−10.4, −0.40]**	−0.83	[−2.62, 0.97]
Sex	**−0.07**	**[−0.13, −0.01]**	**−0.09**	**[−0.15, −0.04]**	0.15	[−0.24, 0.54]
Dominance status	**−0.02**	**[−0.03, 0.00]**	**−0.02**	**[−0.03, −0.00]**	−0.06	[−0.13, 0.01]
Anxiety	**−0.14**	**[−0.26, −0.02]**	**−0.13**	**[−0.23, −0.03]**	−0.32	[−0.83, 0.17]
Dominance status × Anxiety	**0.02**	**[0.00, 0.03]**	**0.02**	**[0.00, 0.03]**	0.04	[−0.02, 0.10]

Bold indicates estimates whose confidence interval did not overlap with 0. We observed rhesus macaques (*Macaca mulatta*) at the Oregon National Primate Research Center between March and June 2014, and at the California National Primate Research Center between January and April 2014

**Table VI:** Mixed models of lower-height/full-height ratio and age variables

Variable	All individuals		Younger		Older	
	*B*	95% C.I.	*B*	95% C.I.	*B*	95% C.I.
Age	**−0.18**	**[−0.3, −0.06]**	−0.29	[−0.81, 0.25]	−0.04	[−1.25, 01.16]
Age^2^	**0.40**	**[0.16, 0.63]**	0.70	[−1.71, 2.98]	0.28	[−1.27, 1.83]
Age^3^	**−0.23**	**[−0.36, −0.1]**	−0.36	[−3.27, 2.74]	−0.21	[−0.82, 0.40]

Bold indicates estimates whose confidence interval did not overlap with 0. We observed rhesus macaques (*Macaca mulatta*) at the Oregon National Primate Research Center between March and June 2014, and at the California National Primate Research Center between January and April 2014

**Table VII:** Mixed models of lower-height/full-height ratio, personality, and age variables

Variable	All individuals		Younger		Older	
	*B*	95% C.I.	*B*	95% C.I.	*B*	95% C.I.
Age	−0.13	[−0.28, 0.04]	−0.15	[−0.72, 0.43]	−0.82	[−2.51, 0.87]
Age^2^	**0.33**	**[0.02, 0.61]**	−1.01	[−3.60, 1.57]	1.24	[−0.98, 3.27]
Age^3^	**−0.20**	**[−0.35, −0.03]**	2.85	[−0.56, 6.27]	−0.54	[−1.32, 0.31]
Confidence	−0.04	[−0.11, 0.03]	**−0.14**	**[−0.23, −0.05]**	0.05	[−0.10, 0.19]
Openness	0.04	[−0.02, 0.10]	−0.01	[−0.10, 0.07]	0.12	[−0.03, 0.25]
Assertiveness	0.03	[−0.05, 0.10]	**0.16**	**[0.07, 0.27]**	−0.05	[−0.20, 0.10]
Friendliness	0.01	[−0.03, 0.04]	0.02	[−0.05, 0.79]	−0.06	[−0.13, 0.03]
Activity	−0.02	[−0.06, 0.03]	−0.04	[−0.09, 0.02]	−0.03	[−0.11, 0.04]
Anxiety	−0.02	[−0.07, 0.03]	−0.05	[−0.15, 0.04]	−0.02	[−0.04, 0.07]

Bold indicates estimates whose confidence interval did not overlap with 0. We observed rhesus macaques (*Macaca mulatta*) at the Oregon National Primate Research Center between March and June 2014, and at the California National Primate Research Center between January and April 2014

**Table VIII:** Summary of results from rhesus macaques (*Macaca mulatta*) observed at the Oregon National Primate Research Center between March and June 2014, and at the California National Primate Research Center between January and April 2014

	Facial width-to-height ratio		Lower-height/full-height ratio	
	Younger	Older	Younger	Older
Sex	−	.	.	.
Sex × Age	.	.	.	.
Confidence	−	.	−	.
Openness	.	.	.	.
Assertiveness	+	−	+	.
Friendliness	.	.	.	.
Activity	−	.	.	.
Anxiety	(−)	(−)	.	.
Dominance status	(−)	(−)	.	.
Anxiety × Dominance status	−	−		

A plus sign indicates a positive relationship, a minus sign indicates a negative relationship, and a dot indicates no significant relationship, all via regression models. Associations between Anxiety and facial width-to-height ratio are in parentheses to indicate that this association was present only when the Anxiety by Dominance status interaction was included
