# Supplementary material for: An Exploration of the Relationships Among Facial Dimensions, Age, Sex, Dominance Status, and Personality in Rhesus Macaques (Macaca mulatta)
Source: Int J Primatol. Author manuscript; Available in PMC 2020 Aug 3. (PMC7398590; doi:10.1007/s10764-019-00104-y)
Supplement: Supplement [file NIHMS1049742-supplement-Supplement.docx]

**Electronic Supplementary Material**

**An Exploration of the Relationships Among Facial Dimensions, Age, Sex, Dominance Status, and Personality in Rhesus Macaques (*Macaca mulatta*)**

**D. M. Altschul · L. M. Robinson · K. Coleman · J. P. Capitanio · V. A. D. Wilson**

**Results**

Reliability of Point Measurements

Each point consists of two coordinates, which are spatially related. Two coordinate observations do not lend themselves to statistics designed for reliability, so for our purposes, we calculated the linear Euclidean distance between the first and second measurement of each point. We then found the mean, median, and 95% confidence interval for each point type (Table SII).

Almost all images in our sample were several thousand pixels high and wide, so the mean and medians are at most about 1% of this, and the high end of the confidence intervals are at most 2%. These values are very good, on par with high intraclass correlation calculations of test–retest reliability.

Preliminary Correlations

Preliminary, untested correlations were calculated for fWHR, fLHFH, age, normalized David’s score (social rank), and all personality metrics (Table SIII).

*Low-Ranking Drivers of fWHR.*

Given that we found positive and negative relationships between fWHR and Assertiveness in younger and older macaques, respectively, we wished to determine if these effects were being driven by individuals on the low end of the rank spectrum. We attempted to use two approaches to investigate this: the Johnson–Neyman technique (Johnson and Fay 1950) and mixture modelling using an expectation–maximization algorithm (Muthén and Shedden 1999). The Johnson–Neyman technique allows one to obtain a range of values for which the predictor variables in a regression model would reject the null hypothesis that there is no difference (Potthoff 1964). In our study, we were interested in whether in a linear model of rank predicting fWHR, a significant effect might be present in the low end of the rank spectrum. The Johnson–Neyman technique’s ability to identify significant ranges of predictor variables might identify this in rank. Mixture modeling would allow us to fit two different linear slopes to different segments of the population, and expectation–maximization algorithmically selects the best place for a cut-point between the two regression slopes. It is possible that one of these slopes might not be significantly different from zero, so in our case, the algorithm might identify a cut-point and regression slope that could indicate that rank predicted fWHR, but only on one end of the rank spectrum, as we wished to test. Unfortunately, our data were not numerous enough to support either of these analyses.

|  | **Table SI** Demographic information for observed macaque groups | | | | |
| --- | --- | --- | --- | --- | --- |
|  |  | | | | |
|  | | Facility | *N* | Males | Mean years old ± SD |
| Group 1 | | ONRPC | 11 | 11 | 4.31 ± 1.21 |
| Group 2 | | ONRPC | 15 | 9 | 3.57 ± 0.64 |
| Group 3 | | ONRPC | 15 | 9 | 3.43 ± 0.69 |
| Group 4 | | CNPRC | 15 | 3 | 8.91 ± 3.56 |
| Group 5 | | CNPRC | 16 | 4 | 8.40 ± 5.61 |
| Group 6 | | CNPRC | 13 | 7 | 6.79 ± 5.54 |

| **Table SII** Reliability derived from linear distance between repeated point measurements | | | | | |
| --- | --- | --- | --- | --- | --- |
|  |  |  |  |  |  |
| Point | Mean | Median | 95% | C.I. |  |
| A | 26.0 | 12.0 | [9.71, | 42.3] |  |
| B | 31.2 | 12.1 | [9.53, | 52.9] |  |
| C | 14.2 | 7.61 | [3.94, | 24.4] |  |
| D | 12.0 | 6.07 | [1.92, | 22.2] |  |
| E | 31.6 | 23.5 | [17.8, | 45.4] |  |
| F | 26.8 | 16.6 | [15.0, | 38.6] |  |
| G | 29.9 | 14.6 | [11.0, | 48.7] |  |
| Measurements were made in pixels. | | | | |  |

| **Table SIII** Correlation matrix of variables of interest | | | | | | |  |  |  |  |  |  |  |
| --- | --- | --- | --- | --- | --- | --- | --- | --- | --- | --- | --- | --- | --- |
|  | 2 | 3 | 4 | 5 | 6 | 7 | 8 | 9 | 10 | 11 | 12 | 13 | 14 |
| 1. fWHR | 0.19 | 0.02 | 0.13 | −0.01 | −0.04 | 0.01 | 0.10 | −0.06 | 0.10 | −0.07 | 0.10 | −0.05 | 0.06 |
| 2. fLHFH |  | −0.06 | 0.01 | −0.05 | 0.02 | 0.00 | 0.09 | −0.07 | 0.12 | −0.03 | 0.07 | 0.04 | 0.04 |
| 3. Dominance status |  |  | 0.20 | 0.79 | 0.50 | 0.81 | −0.51 | 0.80 | 0.44 | 0.85 | 0.48 | 0.26 | −0.17 |
| 4. Age |  |  |  | 0.20 | −0.37 | 0.23 | −0.21 | 0.13 | −0.58 | −0.10 | −0.23 | −0.68 | −0.52 |
| 5. Short.Con |  |  |  |  | 0.55 | 0.91 | −0.62 | 0.98 | 0.36 | 0.87 | 0.61 | 0.13 | −0.37 |
| 6. Short.Opn |  |  |  |  |  | 0.52 | −0.21 | 0.49 | 0.94 | 0.69 | 0.71 | 0.65 | 0.19 |
| 7. Short.Ast |  |  |  |  |  |  | −0.45 | 0.92 | 0.42 | 0.95 | 0.52 | 0.22 | −0.24 |
| 8. Short.Anx |  |  |  |  |  |  |  | −0.68 | 0.18 | −0.28 | −0.53 | 0.45 | 0.90 |
| 9. Confidence |  |  |  |  |  |  |  |  | 0.30 | 0.82 | 0.64 | 0.03 | −0.48 |
| 10. Openness |  |  |  |  |  |  |  |  |  | 0.60 | 0.61 | 0.78 | 0.44 |
| 11. Assertiveness |  |  |  |  |  |  |  |  |  |  | 0.51 | 0.43 | −0.01 |
| 12. Friendliness |  |  |  |  |  |  |  |  |  |  |  | 0.20 | −0.29 |
| 13. Activity |  |  |  |  |  |  |  |  |  |  |  |  | 0.69 |
| Correlations are Spearman’s rank coefficient, *ρ*. Numbered variables along the top of the table correspond to the values printed by the variables along the side; 14 is Anxiety. Rank = Normalized David’s score; Short.Con = short-form Confidence construct; Short.Opn = short-form Openness; Short.Ast = short-form Assertivness; Short.Anx = short-form Anxiety. | | | | | | | | | | | | | |

**Table SIV** Linear mixed models of age and sex on fWHR

| Variable | Age variables  *B* | 95% C.I. | Sex included  *B* | 95% C.I. |
| --- | --- | --- | --- | --- |
| Age | −0.22 | [−0.47, 0.03] | −0.07 | [−0.52, 0.06] |
| Age^2^ | 0.48 | [−0.01, 1.00] | −0.22 | [−0.11, 1.07] |
| Age^3^ | −0.28 | [−0.56, −0.01] | 0.47 | [−0.62, 0.05] |
| Sex |  |  | −0.27 | [−0.13, −0.01] |
| Sex × Age |  |  | 0.03 | [−0.04, 0.11] |

| **Table SV** Mixed models of fWHR and personality as assessed by the short-form items | | | | | | | |  |
| --- | --- | --- | --- | --- | --- | --- | --- | --- |
|  |  | All individuals |  |  | Younger |  |  | Older |
| Variable | *B* | 95% C.I. |  | *B* | 95% C.I. |  | *B* | 95% C.I. |
| Age | −0.22 | [−0.49, 0.03] |  | **−1.02** | **[−1.98, −0.17]** |  | −1.01 | [−4.63, 2.51 |
| Age^2^ | 0.40 | [−0.06, 0.96] |  | 3.65 | [−0.07, 7.85] |  | 1.40 | [−3.24, 6.14] |
| Age^3^ | −0.22 | [−0.53, 0.04] |  | −3.90 | [−9.19, 0.85] |  | −0.59 | [−2.49, 1.26] |
| Sex | **−0.08** | **[−0.14, −0.01]** |  | **−0.09** | **[−0.14, −0.05]** |  | −0.06 | [−0.31, 0.19] |
| Confidence | −0.04 | [−0.11, 0.03] |  | **−0.09** | **[−0.15, −0.03]** |  | 0.09 | [−0.19, 0.38] |
| Openness | −0.02 | [−0.06, 0.02] |  | 0.00 | [−0.03, 0.05] |  | −0.04 | [−0.19, 0.06] |
| Assertiveness | 0.04 | [−0.03, 0.11] |  | **0.06** | **[0.00, 0.12]** |  | 0.02 | [−0.30, 0.23] |
| Anxiety | −0.02 | [−0.06, 0.02] |  | −0.04 | [−0.07, 0.00] |  | 0.07 | [−0.05, 0.19] |

Bold indicates estimates whose confidence intervals do not overlap with 0.

| **Table SVI** Mixed models of fLHFH, age, and personality as assessed by the short-form items | | | | | |  |  |  |
| --- | --- | --- | --- | --- | --- | --- | --- | --- |
|  |  | All individuals |  |  | Younger |  |  | Older |
| Variable | *B* | 95% C.I. |  | *B* | 95% C.I. |  | *B* | 95% C.I. |
| Age | **−0.19** | **[−0.32, 0.07]** |  | −0.34 | [−0.90, 1.59] |  | −0.11 | [−1.30, 1.10] |
| Age^2^ | **0.43** | **[0.18, 0.66]** |  | −0.79 | [−1.80, 3.15] | | 0.37 | [−1.20, 1.89] |
| Age^3^ | **−0.24** | **[−0.37, −0.11]** |  | −0.35 | [−3.48, 2.97] | | −0.23 | [−0.83, 0.39] |
| Confidence | −0.02 | [−0.05, 0.02] |  | −0.02 | [−0.07, 0.02] |  | 0.01 | [−0.07, 0.11] |
| Openness | 0.01 | [−0.02, 0.02] |  | 0.01 | [−0.02, 0.04] |  | 0.00 | [−0.03, 0.04] |
| Assertiveness | 0.02 | [−0.01, 0.05] |  | 0.03 | [−0.01, 0.06] |  | −0.01 | [−0.11, 0.08] |
| Anxiety | 0.00 | [−0.02, 0.02] |  | −0.01 | [−0.03, 0.02] |  | 0.02 | [−0.01, 0.06] |

Bold indicates estimates whose confidence intervals do not overlap with 0.

**References**

Johnson, P. O., & Fay, L. C. (1950). The Johnson–Neyman technique, its theory and application. *Psychometrika*, *15*(4), 349–367.

Muthén, B., & Shedden, K. (1999). Finite mixture modeling with mixture outcomes using the EM algorithm. *Biometrics*, *55*(2), 463–469.

Potthoff, R. F. (1964). On the Johnson–Neyman technique and some extensions thereof. *Psychometrika*, *29*(3), 241–256.
